# Case Report: The molecular profile of granular cell astrocytoma predicts aggressive clinical behavior, independent of morphology

**DOI:** 10.3389/pore.2026.1612388

**Published:** 2026-05-04

**Authors:** Melanie Jensen, Pranoy Das, Oliver Wroe-Wright, Oscar MacCormac, Francesco Marchi, Ali Elhag, Yasir A. Chowdhury, Ross Laxton, Rebecca Ellmers, Omar Al-Salihi, Keyoumars Ashkan, Ranjeev Bhangoo, Francesco Vergani, Istvan Bodi, Zita Reisz, Jose Pedro Lavrador

**Affiliations:** 1 Department of Clinical Neuropathology, King’s College Hospital NHS Foundation Trust, London, United Kingdom; 2 Neurosurgical Department, King’s College Hospital NHS Foundation Trust, London, United Kingdom; 3 Department of Neurosurgery, Neurocenter of Southern Switzerland, Lugano, Switzerland; 4 Molecular Neuropathology, Synnovis at King’s College Hospital NHS Foundation Trust, London, United Kingdom; 5 Department of Neuro-Oncology, Guy’s Cancer Centre, London, United Kingdom

**Keywords:** 5-ALA, aggressive behaviour, DNA methylation profiling, granular cell astrocytoma/glioblastoma, IDH-wildtype

## Abstract

**Background:**

Granular cell astrocytoma (GCA) is a rare, morphologically distinct variant of IDH-wildtype glioblastoma that can appear deceptively low-grade yet behave aggressively. Its molecular features remain poorly defined, and no methylation-based classification has previously been reported.

**Methods:**

Two GCAs diagnosed in our clinical neuropathology department were described with the integration of clinical, intraoperative, histopathological, and molecular data, including DNA methylation profiling, a targeted next-generation sequencing panel, and, in one case, whole genome sequencing (WGS).

**Results:**

The patients were aged 63 and 54 years old, respectively, both presenting with supratentorial tumors showing granular cell morphology. Case 1 showed a densely cellular tumor composed entirely of bland-appearing granular cells without a conventional astrocytic component. Case 2 showed low-grade granular cell areas transitioning into high-grade astrocytic regions with mitoses, microvascular proliferation, and necrosis. Despite these morphological differences, both cases matched the methylation class “Glioblastoma, IDH-wildtype, mesenchymal subtype” and shared molecular features typical of glioblastoma, including chromosome +7/−10 and *CDKN2A/B* deletion. Both patients harbored oncogenic *NF1* variants. WGS in Case 2 also revealed homozygous *MTAP* loss and chromoanasynthesis on chromosome 9. Case 1 received Stupp protocol chemoradiotherapy, recurred after 3 months of treatment, and died 11 months after diagnosis. Case 2 has progressed with a new posterior fossa lesion while on adjuvant temozolomide.

**Conclusion:**

These cases demonstrate that GCAs span a morphological spectrum yet molecularly correspond to the mesenchymal subtype of IDH-wildtype glioblastoma. Integrated molecular testing is therefore essential for accurate diagnosis and for guiding clinical management, including consideration for potential clinical trial enrollment.

## Introduction

Granular cell astrocytoma (GCA) is a rare but aggressive infiltrative glial tumor variant recently recognized as a morphological subtype of *IDH*-wildtype glioblastoma in the 5th edition of the WHO Classification of Central Nervous System (CNS) Tumors [1]. Histologically, GCA is characterized by large tumor cells containing abundant eosinophilic, periodic acid–Schiff (PAS)-positive cytoplasmic granules, which ultrastructurally correspond to the accumulation of lysosomes within the cytoplasm [2]. While many cases exhibit a conventional astrocytic component, some display a deceptively low-grade morphology composed purely of granular cells. In certain cases, a prominent lymphocytic infiltrate may be present, further complicating the distinction from other neoplastic or non-neoplastic CNS lesions [2]. Despite this histological variability, GCAs typically demonstrate aggressive clinical behavior, underscoring the importance of early and accurate diagnosis to guide optimal management, including maximal safe resection and adjuvant therapy. However, molecular data on GCA remain limited, with few reports describing recurrent or targetable genetic alterations [3–5] and methylation profiling studies have not yet been reported. To address this gap, we conducted a comprehensive literature review using the PubMed database to identify relevant publications on GCA and extracted data that included patient demographics, histopathological characteristics, molecular findings, and clinical outcomes. The principal published reports are summarized in Supplementary Table S1. In addition, we present the first reported cases of GCA evaluated with intraoperative 5-aminolevulinic acid (5-ALA) fluorescence and DNA methylation profiling. This combined clinicopathological and molecular approach provides new insights into the diagnostic and biological features of this rare tumor subtype.

## Case reports

### Case 1

**FIGURE 1:**
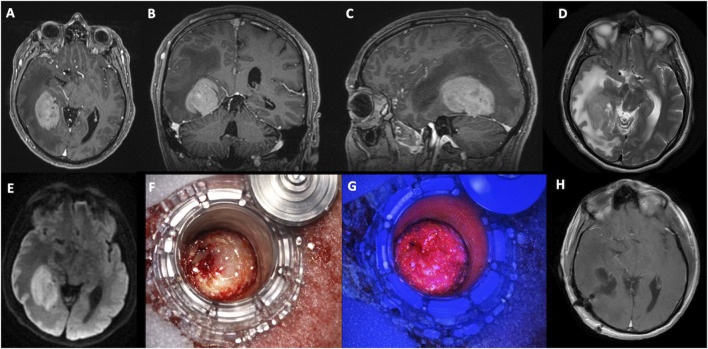
Case 1 right posterior fusiform lesion with homogenous contrast enhancement and significant associated edema causing significant mass effect and uncal herniation – preoperative T1-weighted images with Gadolinium in the axial **(A)**, coronal **(B)**, and sagittal **(C)** planes. **(D)** shows the preoperative T2-weighted image documenting significant perilesional edema. **(E)** (diffusion-weighted image) shows intense restriction to diffusion of water molecules consistent with a high cellular lesion. **(F,G)** show intraoperative images of tubular retractor-assisted resection with the BrainPath Stryker®, with significant 5-Aminolevulinic Acid (5-ALA) fluorescence under the BLUE 400 filter using the Kinevo Zeiss® Microscope. **(H)** (postoperative T1-weighted images with Gadolinium in the axial plane) shows complete tumor resection.

A 63-year-old man with no significant past medical history presented to the emergency department with a 1-week history of confusion, left-sided hemiparesis, and right-sided hemianopia. A CT scan of the head showed an isodense right temporo-occipital intrinsic lesion with surrounding edema and a significant mass effect. An MRI confirmed a 6.5 cm mass in the right temporo-parietal region with homogeneous contrast enhancement, diffusion restriction, and hyperintensity on T2/FLAIR, with 14 mm of leftward midline shift and marked perilesional edema causing ongoing mass effect (Figures 1A–E). A CT scan of the chest, abdomen, and pelvis (CAP) did not show any primary tumor. Given the main differential diagnoses discussed at the neuro-oncology multidisciplinary meeting (primary CNS lymphoma versus high-grade glioma), the patient underwent an image-guided burr hole biopsy, which, alongside the intraoperative smear findings, supported a diagnosis of GCA. Given the presence of a glial tumor with a significant mass effect in an otherwise healthy patient, tumor resection was undertaken. A trans-sulcal, minimally invasive parafascicular approach (MIPS) was performed with the aid of preoperative tractography, intraoperative neuromonitoring, ultrasound, and 5-ALA guidance. (Figures 1F,G). An extradural slip electrode was inserted, and baseline somatosensory, motor-evoked, and visual-evoked potentials (SSEPs, MEPSs, and VEPs) were measured. The macroscopic appearance of the tumor was pale grey, and it fluoresced avidly under ultraviolet light. The superior margin of the resection was limited by a drop in VEPs to 20% of the baseline. The extent of the resection was guided anteriorly by fluorescence, superiorly by the lateral ventricle, and inferiorly by the temporo-occipital arachnoid and the limit of fluorescence (Figure 1G). A post-operative MRI scan showed no residual enhancing tumor at the T1 contrast sequences, consistent with gross total resection (Figure 1H). Following surgery, the patient’s recovery was slow, with reduced mobility and a requirement for assistance with activities of daily living. The patient was treated with dexamethasone 2 mg once daily for tumor-associated cerebral edema, which was later converted to prednisolone due to a steroid-related rash. During disease progression, escalation of steroid therapy (to up to 85 mg of prednisolone daily) was associated with transient symptomatic improvement, although this effect diminished over time. The patient received the standard Stupp protocol of chemoradiotherapy, comprising 60 Gy in 30 fractions with concurrent temozolomide (145 mg daily), followed by five cycles of adjuvant temozolomide. He was also enrolled in the TRIDENT trial and received tumor-treating fields (Optune), which were discontinued approximately 3 months after completion of chemoradiotherapy due to radiological progression. Early post-treatment imaging, performed within weeks of completing chemoradiotherapy, raised the possibility of pseudoprogression, as the changes occurred within the radiotherapy field and within the expected temporal window. However, on short-interval follow-up imaging, there was progressive solid nodular enhancement with diffusion restriction and increasing lesion size, fulfilling RANO criteria for true disease progression, as confirmed at neuro-oncology multidisciplinary review. The recurrence was centered at the margins of the resection cavity, with subsequent ependymal extension and, over the following months, development of leptomeningeal dissemination involving the upper cervical spinal cord and cranial nerve complexes was observed. In the context of ongoing clinical and radiological deterioration, re-irradiation was not pursued. Following further progression, it was agreed that no further active oncological treatment would be offered, as this was unlikely to improve quality of life. The patient was transitioned to specialist palliative care and died within approximately 1 month of cessation of active treatment and 11 months post-diagnosis.

#### Pathology and molecular findings

**FIGURE 2:**
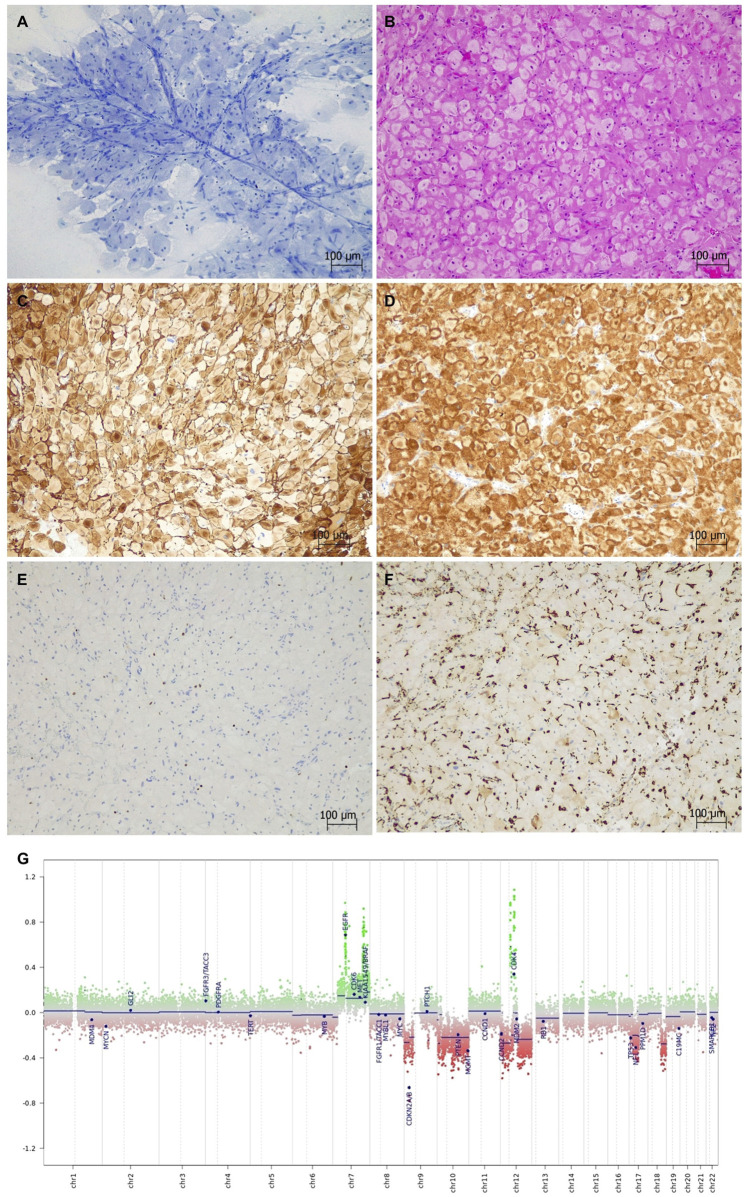
Case 1 granular cell astrocytoma/glioblastoma (Toluidine blue stain and Hematoxylin-eosin stain). **(A,B)** Monotonous histiocyte-like cells with abundant finely granular cytoplasm and mildly atypical nuclei are intermingled with thin-walled blood vessels. **(C,D)** The neoplastic cells are strongly positive with GFAP and EMA. **(E)** Olig2 labels only a few scattered tumor cells. **(F)** CD68 is negative in the majority of the tumor. **(G)** A copy number variation plot generated by a DNA methylation array shows chromosome +7/−10 in addition to *EGFR*, 7q33, and *CDK4* amplification and homozygous *CDKN2A/B* deletion.

An intraoperative smear of the fresh tumor specimen revealed a cellular lesion consisting of large histiocyte-like cells with abundant, faintly Toluidine-blue granular cytoplasm and mildly atypical, often eccentric, nuclei. These cells were radially arranged around thin blood vessels and showed a fairly monotonous pattern in four different smear specimens, with no mitotic activity, necrosis, or significant inflammation in the background (Figure 2A). There was no evidence of transition into a conventional astrocytic component. Based on these features, the possibility of a granular cell glioma was raised, while an unusual histiocytic tumor was considered less likely. The radiological features and the absence of inflammatory cells and necrosis did not support other histiocyte-rich lesions, such as ischemic infarction or demyelination.

Histological sections confirmed a densely cellular intrinsic neoplasm with compact sheets of monomorphic, rounded cells exhibiting a finely granular, eosinophilic cytoplasm, often with a ring-like, perimembranous pattern, most closely resembling the features of a granular cell tumor (Figure 2B). A single prominent blood vessel was noted in addition to focal perivascular lymphocyte cuffing, but mitotic figures and necrosis were not present in the sample. No definite microvascular proliferation was identified. An Alcian blue/Periodic acid-Schiff (PAS) stain highlighted a few cells with PAS-positive cytoplasmic granules. Immunohistochemical analysis showed intense GFAP, S100, and EMA positivity in the tumor, while Olig2 labeled only a few scattered neoplastic cells (Figures 2C–E). The majority of the tumor was negative for histiocytic/lysosomal markers, such as HLA-DR and CD68 (Figure 2F), arguing against a histiocytic origin. There was no immunoreactivity for the IDH1 (R132H) mutant clone, and ATRX expression was retained. The p53 immunostaining showed strong nuclear positivity across the tumor (mutant pattern). The Ki-67 proliferation index focally reached 4%–5%. Despite its deceptively low-grade cytological appearance, the immunophenotype, granular cell morphology, and patient age strongly supported an IDH-wildtype glioblastoma in the integrated diagnostic context; therefore, a provisional diagnosis of granular cell astrocytic glioma was made pending molecular studies.

A DNA methylation array (Illumina MethylationEPIC 850k; DKFZ Brain tumor methylation classifier v12.5) revealed a strong match for “MC Glioblastoma, IDH-wildtype, mesenchymal subtype” with a calibrated score of 0.99. Next-generation sequencing (NGS - Qiagen QIAseq Multimodal Panel assessing a targeted DNA panel of 305 genes and an RNA panel of 76 genes) found an oncogenic nonsense *NF1* variant and a likely oncogenic missense variant in *TP53*. The tumor was *IDH-* and *TERT* promoter*-*wildtype, and no actionable fusion was identified. Copy number variation (CNV) analysis using a methylation array and an NGS CNV kit revealed multiple structural abnormalities, including chromosome 7 gain and 10 loss (+7/-10), amplification of the *EGFR*, *CDK4,* and *DDIT3* genes, and homozygous deletion of *CDKN2A/B* (Figure 2G). There was also loss of the *TP53*, *ATRX,* and *NF1* genes in addition to loss of heterozygosity (LOH) in chromosomes 7, 10, and 17p. The *MGMT* gene promoter was unmethylated (1%) by pyrosequencing (Molecular methods are provided in the Supplementary Materials). In view of the molecular profile of the tumor, the final integrated diagnosis was “Granular cell glioblastoma, IDH-wildtype, mesenchymal subtype (methylation-based), CNS WHO grade 4”.

### Case 2

**FIGURE 3:**
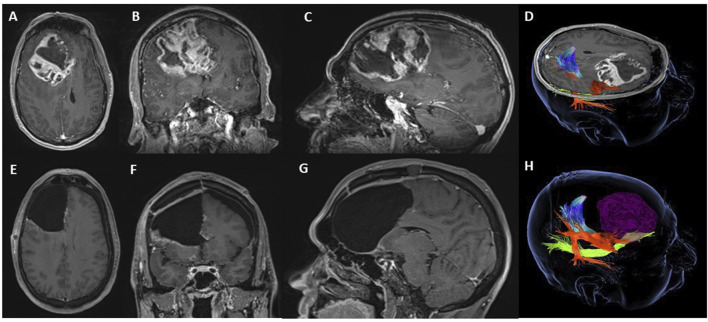
Case 2 right frontal contrast-enhancing lesion with a mixed cystic-necrotic component and invasion of the genu of the corpus callosum with minimal extension to the contralateral hemisphere and ipsilateral ependymal invasion causing a midline shift – preoperative T1-weighted images in the axial **(A)**, coronal **(B)**, and sagittal **(C)** planes. **(D,H)** show 3D models of the tumor and its relation to the subcortical white matter structures of interest: the corticospinal tract (multicolor), the arcuate fasciculus (orange), and the inferior fronto-occipital fasciculus (green). The Uncinate Fasciculus and Fronto-Aslant Tract were not reliably dissected using the constrained spherical deconvolution algorithm (Medtronic S8®). Postoperative images at 1 month of follow-up document complete resection of the contrast-enhancing component of this tumor with no complications - postoperative T1-weighted images in the axial **(E)**, coronal **(F)**, and sagittal **(G)** planes.

A 54-year-old man presented to his local Accident & Emergency Department with symptoms indicative of increased intracranial pressure, including a 2–3 weeks history of fatigue, unsteadiness, vomiting, confusion, and intermittent headaches with minimal response to routine analgesics. An MRI of the head revealed a large, heterogeneous space-occupying lesion centered in the right frontal lobe, which was causing a significant mass effect on the right ventricle and supratentorial brain. The lesion extended across the midline to the left side, resulting in a 9.2-mm midline shift, brain herniation, and brainstem crowding (Figures 3A–D,H). The patient was commenced on dexamethasone 4 mg twice daily for 3 days pre-operatively to reduce tumor-associated vasogenic edema and optimize neurological status. Following surgical resection of the contrast-enhancing portion of the tumor, the patient’s symptoms improved, and he was discharged on postoperative day 3 without any focal neurological deficits (Figure 3G). Following surgical resection, the patient received standard Stupp protocol chemoradiotherapy (60 Gy in 30 fractions with concurrent temozolomide), followed by adjuvant temozolomide administered over three cycles (150–200 mg/m^2^ on days 1–5 of a 28-day cycle). Interval imaging approximately 4–5 months post-operatively demonstrated stable appearances at the primary resection site but revealed a new enhancing lesion in the left cerebellar dentate region. Temozolomide was therefore discontinued. The case was discussed at a multidisciplinary team meeting, and the lesion was deemed unamenable to surgical resection. The patient underwent focal re-irradiation to the posterior fossa (40 Gy in 15 fractions). The patient was also assessed for clinical trial enrollment but was not eligible due to radiological disease progression in a high-risk location with concerns about fourth ventricular compression. Following re-irradiation, the patient experienced a gradual clinical decline with increasing fatigue and worsening mobility but remains alive and under ongoing clinical follow-up at the time of writing (approximately 1 year after initial presentation).

#### Pathology and molecular findings

**FIGURE 4:**
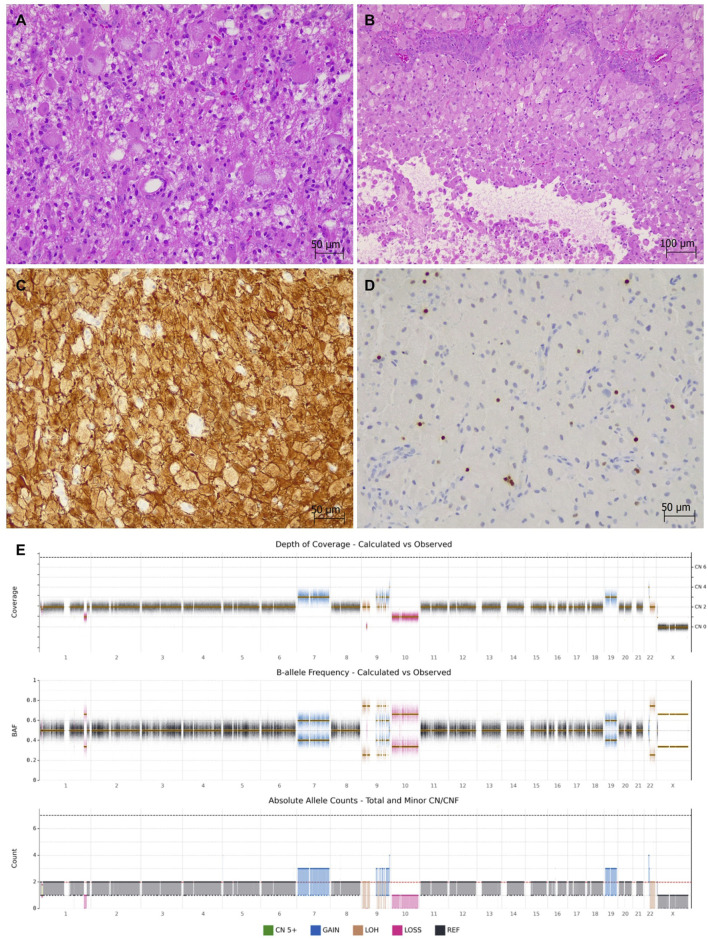
Case 2 granular cell glioma with high-grade features (Hematoxylin-eosin staining). **(A,B)** Transition of the granular cell component into conventional astrocytic morphology. Foci of necrosis and florid microvascular proliferation. **(C)** Strong GFAP expression. **(D)** Rare Olig2-positive cells. **(E)** Structural variant analysis by whole genome sequencing revealed chromosome +7/−10, segmental gains of chromosomes 9q and 22q, segmental loss of chromosome 1q, and homozygous loss of *CDKN2A/B* and *MTAP*. A chromoanasynthesis event was detected on chromosome 9.

Intraoperative smears and histological sections confirmed a hypercellular glial tumor comprising sheets and nests of markedly atypical, elongated cells in a fibrillary matrix, which is typical of a conventional astrocytic tumor. However, there were also tumor regions composed of sheets of monomorphic, round-to-polygonal cells with abundant, finely granular eosinophilic cytoplasm and distinct cell borders, in keeping with a granular cell component. Mitotic figures, microvascular proliferation, and necrosis were identified (in keeping with a high-grade tumor), in addition to perivascular lymphocyte cuffing (Figures 4A,B). There was also evidence of leptomeningeal invasion. The immunoprofile of the tumor was similar to Case 1, with widespread GFAP positivity, very focal Olig2 expression, and no immunoreactivity for the IDH1 (R132H) mutant clone (Figures 4C,D). The Ki-67 proliferation index was high (15%–20%). These features favored a molecularly defined IDH-wildtype glioblastoma, given the age of the patient.

A DNA methylation array (using Brain classifier v12.8) profiled the tumor as “Glioblastoma, IDH-wildtype, mesenchymal subtype” with a calibrated score of 0.98. NGS found an oncogenic *TERT* promoter variant along with two *NF1* variants (one nonsense and one missense). There were multiple structural abnormalities detected on the CNV plot, including chromosome +7/−10, segmental gains of chromosomes 9q and 22q, segmental loss of chromosome 1q, and homozygous loss of *CDKN2A/B*. The *MGMT* promoter was unmethylated (0.9%). Whole genome sequencing (WGS) confirmed three domain 1 variants through standard-of-care testing, in addition to identification of homozygous *MTAP* loss, an out-of-frame *PTEN* fusion, and a chromoanasynthesis event on chromosome 9 (Figure 4E). No germline variants were identified. Altogether, the histo-molecular features were consistent with “Granular cell glioblastoma, IDH-wildtype, mesenchymal subtype (methylation-based), CNS WHO grade 4”.

## Discussion

The first description of a granular cell tumor in humans was provided by Abrikossoff in 1926, who referred to it as a “myoblastoma of the tongue”. Since then, granular cell tumors have been identified in various extracranial locations, including the gastrointestinal and urinary tracts, skin, and soft tissues. Within the CNS, granular cell tumors are typically derived from the neurohypophyseal glia, most commonly at the infundibulum, and are generally benign, non-infiltrative lesions [6].

In contrast, the first granular cell astrocytoma (GCA), described by Markesbery in 1973, exhibited distinctly aggressive features, including infiltrative growth and malignant behavior, differentiating it from the granular cell tumors of the neurohypophysis [7]. The term “Granular cell astrocytoma” was formally introduced by Brat et al. in 2002, who also presented the largest case series to date. This detailed the histopathological features, grading, and clinical outcome of this entity [2]. Just over 100 cases of GCA have been reported thus far, with different levels of clinical and pathological information [5, 8]. These tumors most often involve the parietal lobe, although they have also been described in the pineal gland, cerebellum, corpus callosum, basal ganglia, and central canal [1]. GCAs show a male predominance (male-to-female ratio of approximately 2:1) in a population with a wide age distribution, ranging from young adults to individuals over 80 years old. Typically, they present with seizures, headaches, vomiting, confusion, and focal neurological deficits, depending on tumor location. The prognosis is variable but overall poor (a mean survival of under 1 year), and comparable to that of conventional IDH-wildtype glioblastoma [2, 5]. Given their aggressive course, the current standard of care consists of maximal safe resection with adjuvant chemo/radiotherapy [9]. This clinical heterogeneity underscores the need for standardized diagnostic and prognostic criteria. DNA methylation profiling may offer valuable insight in this respect, potentially clarifying whether GCA represents a distinct biological entity or a spectrum of pathologies currently grouped under a single classification in the current WHO classification. The principal published reports of GCA are summarized in Supplementary Table S1, which demonstrates uniformly poor outcomes despite variable histological appearances. Although further isolated case reports have been described, molecular data remain limited. Notably, none of these reports have included DNA methylation classification or WGS, which represents a key advance of this study.

### Histomorphological features with emphasis on differential diagnosis

GCA is a rare but recognized morphological variant within IDH-wildtype glioblastomas, as noted in the 5th edition of the WHO Classification of CNS Tumors (2021) [1]. Large tumor cells with abundant PAS-positive, diastase-resistant granular cytoplasm may either be scattered throughout the tumor or dominate the histological picture, sometimes obscuring conventional astrocytic features [2]. The granular cell phenotype reflects lysosomal accumulation and can mimic histiocytic cells or macrophages, particularly in the presence of perivascular inflammation, raising the possibility of various histiocytoses (e.g., CNS Rosai-Dorfman disease or Erdheim-Chester disease), a brain infarct, or demyelinating processes [9]. In our first case, bland granular morphology predominated, with no obvious conventional high-grade glioma cells. In contrast, our second case exhibited a biphasic appearance, with granular cells and classic high-grade astrocytic areas, a pattern seen in approximately half of the cases reported in the literature [2, 3, 5]. This morphologic spectrum reinforces the concept that GCA is a histological variant of IDH-wildtype glioblastoma, rather than a distinct clinicopathological entity. Our cases exemplify both extremes of this spectrum and highlight the diagnostic challenges posed by granular cell-dominant tumors, particularly when working with limited biopsy material or intraoperative smears.

Immunohistochemically, GCAs typically express GFAP and S100, consistent with astrocytic differentiation; however, they often show atypical patterns that can complicate diagnostic interpretation. Olig2, a key transcription factor associated with glial lineage commitment, is frequently reduced or absent in granular cell variants [5]. In our first case, granular cell morphology was accompanied by strong GFAP and S100 expression, focal EMA reactivity, and only sparse Olig2 staining; these features mirror those reported by Brat et al. and, more recently, by Vizcaíno et al. [2, 5]. The tumor was negative for CD68 and HLA-DR, arguing against a histiocytic lesion. Interestingly, the WHO notes that granular tumor cells may occasionally express macrophage markers, such as CD68, likely reflecting their abundant lysosomal content [1]. However, expression of CD163 (a more specific marker of the monocyte–macrophage lineage) is typically absent, supporting a non-histiocytic origin. Our second case showed a similar immunoprofile with slightly more robust GFAP expression and a higher Ki-67 labeling index (∼20%), consistent with its overt high-grade glial morphology.

A lack of correlation between histological grade or Ki-67 indices and clinical outcome in GCAs has been reported previously: in one series, no significant association was found between survival and Ki-67 index [5]. Our findings reinforce this and caution against using morphological features or proliferation index as evidence of indolent behavior in these tumors. Case 1 illustrates this particularly well: although the tumor lacked mitotic activity, necrosis, and definite microvascular proliferation, the integrated histomolecular profile was entirely in keeping with glioblastoma, IDH-wildtype, CNS WHO grade 4. In this setting, vascular prominence alone should not be equated with microvascular proliferation or grade 4 designation, as similar appearances may be encountered in other tumor types, including pilocytic astrocytoma, high-grade astrocytoma with piloid features, pleomorphic xanthoastrocytoma, and ependymoma, and therefore require interpretation in the full clinicopathological and molecular context.

### Molecular characteristics of GCAs: DNA methylation profiling, copy number variation, and recurrent oncogenic variants

To date, molecular studies consistently support the inclusion of GCAs within the spectrum of *IDH*-wildtype glioblastomas [1, 5]. While methylation-based classification is now integrated into the diagnostic algorithm of the WHO CNS 5th edition, this has not previously been reported for granular cell variants. Brain classifier v12.5 and v12.8 (DKFZ) placed both of our reported tumors firmly within the mesenchymal subtype of IDH-wildtype glioblastoma with calibrated scores of 0.99 and 0.98, respectively. The genomic profiles included canonical glioblastoma alterations, such as gain of chromosome 7 and loss of chromosome 10 (+7/–10), homozygous deletion of *CDKN2A/B*, and additional changes, including a *TP53* variant and amplification of the *EGFR*, *CDK4,* and *DDIT3* genes (Case 1), and a *TERT* promoter mutation (Case 2). These findings mirror those in previous case reports and case series, in which *TERT* promoter mutations and +7/–10 copy-number changes were seen in the majority of cases [4, 5]. Interestingly, *NF1* alterations were identified in both cases; each was predicted to result in a premature stop codon and a truncated, non-functional protein. In Case 1, this was accompanied by additional *NF1* loss. These findings are consistent with the reported enrichment of *NF1* alterations within the mesenchymal subclass of glioblastoma [10]. Neither tumor harbored *IDH1*/*2* or *ATRX* mutations, and both lacked *MGMT* promoter methylation, correlating with a poor response to alkylating agents and reduced overall survival [11].

### Clinical behavior and therapeutic options

As previously mentioned, survival in GCAs appears to be poor regardless of histological characteristics. Schittenhelm and Psaras reviewed 59 cases and reported median survival times of 9–11 months, regardless of the presence of high-grade features on histology [12]. Vizcaíno et al. similarly reported a mean overall survival of just 11.3 months in a cohort of 39 patients with no survival correlation to grade, Ki-67 index, or extent of granular cell change [5]. These observations suggest that grading based solely on histology may underestimate the clinical aggressiveness in GCAs. Our cases further support this, particularly Case 1, which lacked overt high-grade features, yet it demonstrated a full molecular profile of glioblastoma with multiple gene amplifications and recurred rapidly despite all therapeutic efforts (resulting in an overall survival of 11 months).

This raises the important question of how survival outcomes might be improved for this tumor variant. From a surgical perspective, optimization of the extent of resection remains a key consideration, and enhanced intraoperative visualization could play a role in achieving this goal. To date, no published data are available regarding the use of 5-ALA fluorescence in GCAs. In Case 1, intraoperative 5-ALA administration resulted in bright, homogeneous fluorescence, a pattern more commonly seen in CNS WHO grade 4 gliomas and less frequently in lower-grade gliomas [13, 14]. However, 5-ALA should not be interpreted as a tool for formal tumor grading. Rather, its established role is as an adjunct for intraoperative visualization of tumor tissue and optimization of the extent of resection.

Regarding adjuvant therapy, temozolomide did not appear to halt tumor progression in either case, likely reflecting the unmethylated *MGMT* promoter status. Nevertheless, a growing number of clinical trials across Europe targeting progressive or recurrent IDH-wildtype glioblastomas may offer potential opportunities for personalized treatment in the near future. For instance, patients with biallelic *NF1* loss may be eligible for MEK inhibitor–based trials, such as the 5G-RUBY study (as in Case 1) [15], while those with homozygous *MTAP* deletion may benefit from emerging *PRMT5* inhibitor trials (as in Case 2) [16].

## Conclusion

These cases highlight that GCAs exhibit diverse morphologies but consistently align with the mesenchymal subtype of IDH-wildtype glioblastoma through methylation profiling. Comprehensive molecular testing is crucial for accurate diagnosis, prognostication, clinical management, and trial eligibility. Methylation analysis, in particular, may enhance diagnostic precision and therapeutic decision-making.

## Data Availability

The original contributions presented in the study are included in the article/Supplementary Material, further inquiries can be directed to the corresponding author.
