# Supplementary material for: Case Report: The molecular profile of granular cell astrocytoma predicts aggressive clinical behavior, independent of morphology
Source: Pathol Oncol Res. 2026 May 4;32:1612388. doi: 10.3389/pore.2026.1612388 (PMC13180715; doi:10.3389/pore.2026.1612388)
Supplement: Supplementary file 1 [file Supplementaryfile1.docx]

**SUPPLEMENTARY MATERIAL 1**

**METHODS**

**Supplementary Table 1.** Antibodies, manufacturers, and dilutions used for immunohistochemistry

| **Antibody** | **Manufacturer** | **Dilution** |
| --- | --- | --- |
| GFAP | Dako (Z0334) | 1:1000 |
| OLIG2 | Abcam (EPR2673) | 1:50 |
| S100 | S-100 (EP32) | Ready to use (Leica) |
| EMA | Dako (E29) | 1:150 |
| IDH1 (R132H) | Dianova (DIA-H09) | 1:400 |
| ATRX | Atlas Antibodies (HPA001906) | 1:300 |
| p53 | Dako (DO-7) | 1:200 |
| CD68 | Dako (M 0876) | 1:50 |
| HLA-DR | Dako (M0775) | 1:300 |
| Ki67 | Dako (M7240) | 1:150 |

**MOLECULAR STUDIES**

1. **MGMT promoter pyrosequencing**

Methylation status of the O6-methylguanine DNA methyltransferase gene (*MGMT*) promoter at four CpG sites was assessed by pyrosequencing technology and the therascreen MGMT pyro kit (Qiagen). Sequence analysed: YGAYGTTYGTAGGTTTTYGT. Sample was bisulphite converted using the Zymo EZ DNA Methylation™ Kit (D5001 or D5002) prior to preparation with the therascreen MGMT pyro kit. An average of the % methylation at four CpG sites was used to determine methylation status. Interpretation was the following: Unmethylated: 0 - 4.99% Borderline methylated:5 - 9.99% Methylated: ≥10%.

1. **DNA Methylation Array**

DNA and RNA extraction: AllPrep DNA/RNA/miRNA Universal Kit and the AllPrep DNA/RNA FFPE Kit were used on the QIAcube Connect MDX. DNA and RNA were extracted from both Fresh Frozen and Formalin-fixed paraffin embedded (FFPE) samples. Sample was bisulphite converted using the Zymo EZ DNA Methylation ™ Kit (D5001 or D5002). Bisulphite treated sample was processed using the Illumina Infinium Methylation EPIC Kit which analyses 850k methylation sites (as per manufacturer's protocols). Diagnostic methylation array testing was performed using the Illumina Infinium HD Methylation Array. Sample was scanned on an Illumina iSCAN. Data was processed via a locally hosted version of the Molecular Neuropathology (MNP) classifier developed by the German Cancer Research Centre in Heidelberg (www.molecularneuropathology.org/mnp)¹. Methylation class calibration score >0.9 was reported with high confidence. CNVs were called by the conumee v1.0 package² and visual inspection. Log2 values +0.15 to +0.4 indicated gains, values >+0.4 indicated amplification. log2 values -0.15 to -0.4 indicated loss, values <-0.4 indicated homozygous loss.

*References:*

1. Capper, David, et al. "Practical implementation of DNA methylation and copy-number-based CNS tumor diagnostics: the Heidelberg experience. "Acta neuropathologica 136.2 (2018): 181-210;

2. Hovestadt V, Zapatka M. conumee: Enhanced copynumber variation analysis using Illumina DNA methylation arrays. R package version 1.9.0, http://bioconductor.org/packages/conumee/;

3. Capper, D. et al (2018). DNA methylation-based classification of central nervous system tumours. Nature, 555: 469 474

1. **DNA and RNA-Fusion Targeted sequencing**

Diagnostic testing of a panel of genes associated with the relevant clinical phenotype described in the national genomic test directory indication, for patients meeting the eligibility criteria (national-genomic-test directory-cancer-2021-22-v2-december-2021).

1. Testing of the indicated gene(s) was performed by the Qiagen QIAseq Multimodal Panel, using single primer extension with unique molecular indexes to assess a targeted DNA panel of 305 genes and a RNA panel of 76 genes associated with solid tumours. Sample was sequenced on an Illumina NextSeq2000, 2X150bp reads with a minimum coverage of 400x (minimum of 6 reads to make a mutation call). Variants at <2% were excluded from analysis and variants at <5% were not considered clinically significant and were therefore not reported. Read alignment and variant calling was performed by BWA-MEM, Samtools, Picard and Vardict followed by BcfTools for variant filtering. Gene fusion analysis was performed using Star-fusion and Arriba.
2. LOH: The heterozygosity of 45 SNVs were plotted and assessed across the 1p and 19q chromosomal regions as per Dubbink et al 2016. Only 1p/19q LOH, LOH of chr 1, 7, 10, 11, 17, 19 and large-scale amplifications/deletions were assessed.
3. Variants were called against genome-build hg19 for the DNA and genome-build hg38 for the RNA. All nomenclature described using the Human Genome Variation Society (HGVS) guidelines (www.hgvs.org). Numbering starts with c.1 at the A of the ATG start codon. Variants were classified using the ACMG/AMP guidelines (Richards et al. 2015) and ACGS best practice guidelines for variant classification in rare disease ([www.acgs.uk.com/guidelines-for-variant-classification-v4-01-2020](http://www.acgs.uk.com/guidelines-for-variant-classification-v4-01-2020)).

*Reference:*

Dubbink, H. J., et al. (2016). Molecular classification of anaplastic oligodendroglioma using next-generation sequencing: a report of the prospective randomized EORTC Brain Tumor Group 26951 phase III trial. Neuro-Oncology 18(3) 388-400

1. **Whole Genome Sequencing**
2. DNA sequencing is carried out in the Illumina Sequencing laboratory Cambridge, and is processed through the Genomics England bioinformatics pipeline. Germline variants are subtracted from the somatic sample to allow analysis of the somatic variants in isolation.
3. Small somatic variants are classified according to the AMP and ACGS 2020 guidelines (acgs.uk.com; https://dx.doi.org/10.1016%2Fj.jmoldx.2016.10.002). Reportable genes are in one of two groups: Domain 1 gene variants are ascribed as potentially actionable (genes in which small variants have reported diagnostic, therapeutic, prognostic or clinical trial associations, as defined by the GenomOncology Knowledge Management System). Domain 2 variants occur in 536 genes which have been causally implicated in cancer, as defined by the Cancer Gene Census (Wellcome Trust Sanger Institute). Variants found in Domain 3 genes (all other genes), are not reported, as these genes are not known cancer-related genes. Small somatic variants are reported if they occur in cancer-specific genes in Domain 1 or 2. Also, CNVs or SVs with breakpoints that overlap genes currently ascribed with potential actionability are reported.
4. Germline variants are reported by Genomics England as either Tier 1 or Tier 3. Tier 1 includes variants deemed to be pathogenic or likely pathogenic which are in cancer susceptibility genes relevant to the disease type. Germline variants are classified according to the ACGS 2020 guidelines (uk-practice-guidelines-for-variant-classification-v4-01-2020.pdf; acgs.uk.com).
5. Variant calls are filtered according to quality and quantity of reads. QC metrics and the filters used in this analysis can be found in the Cancer Genome Analysis Guide.
