# Supplementary material for: Case Report: The molecular profile of granular cell astrocytoma predicts aggressive clinical behavior, independent of morphology
Source: Pathol Oncol Res. 2026 May 4;32:1612388. doi: 10.3389/pore.2026.1612388 (PMC13180715; doi:10.3389/pore.2026.1612388)
Supplement: Supplementary file 2 [file Supplementaryfile2.docx]

Supplementary Table S1. Summary of published series of granular cell astrocytoma

| **Paper (year)** | **N (patients)** | **Clinical / radiological** | **Pathology** | **Molecular** | **Survival / outcome** |
| --- | --- | --- | --- | --- | --- |
| **Vizcaino et al., “Granular cell astrocytoma…” 2018** | 39 | Median age 57.8; mainly frontal/temporal; WHO II (14), III (11), IV (14) | Granular cell morphology diffuse (31/39) and partial (8/39) GFAP+ (28/31), OLIG2+ (16/16), CD68+ (27/30); other histiocytic markers negative (22/22) IDH1 R132H negative (16/16); ATRX retained (12/12) | Cytogenetics: monosomy 10 (6/6), +7 (4/6), −13q (4/6), −14 (4/6)  Next generation sequencing: *PTEN*/*PIK3* (6/13), *NF1* (3/10), *TP53* (3/13), *PALB2* (3/10), *STAG2* (3/10), *EGFR* mutation/amp (3/13), *AR* (2/10), *CDKN2A*/B del (5/13), *TERT* C228T (9/13) | Mean OS 11.3 months; age >60 associated with worse survival; no survival difference by WHO grade or extent of granular change |
| **Brat et al., “Infiltrative Astrocytomas With Granular Cell Features…” 2002²** | 22 | Age 29–75; 17 men, 5 women; common symptoms: seizures, headache, aphasia, hemiparesis; high-grade tumours = contrast-enhancing hemispheric masses with marked oedema | Sheets of PAS-positive granular cells; transition to typical infiltrating astrocytoma in 16/22 4 grade 2, 7 grade 3, 11 grade 4 GFAP+ in all but one; frequent S100, KP1, ubiquitin, EMA+; Ki67 variable | Not performed | 15/18 recurred and died; mean survival 7.6 months; 1 alive at 51 months |
| **Castellano-Sanches et al., “Granular cell astrocytomas show high frequency of allelic loss…” 2003** | 11 | 7 men and 4 women, ages 46-75 years; tumours located in frontal (4), temporal (3), parietal (3), and occipital lobes (1) | 30-100% granular cell component; 3 cases grade 2, 1 case grade 3, 6 cases grade 4 | Higher frequencies of LOH at 1p, 9p, 10q, 17p, and 19q than typical infiltrating astrocytomas of similar grades Losses on 9p and 10q in nearly all cases, including low-grade lesions *TP53* mutations in 2 grade 4 cases Combined p14ARF and p16 (*CDKN2A*) homozygous deletions in 1 grade 4 case. No EGFR amplification; no genetic alterations specific for GCA | 8 cases recurred and died within 1 year; 1 case no recurrence at 51 months; 2 cases follow-up unavailable |
| **Geddes et al., “Granular cell change in astrocytic tumours” 1996** | 5 | 3 women and 2 men, ages 55-66 years; tumours parietal (2) parieto-occipital (1) tempero-parietal (1) frontal (1) | Variable transition to astrocytic component; GFAP positive in 4/5 cases; S100 positive in 4/5 cases; grading not explicitly mentioned | Not performed | 4 cases died within 4-8 months of presentation; 1 case alive at 9-months follow-up |
| **Senetta et al., “Mesenchymal/radioresistant traits in granular astrocytomas” 2016³** | 3 | 68-year-old male, 41-year-old female, 56-year-old male; tumours in temporal lobe (2) and occipital lobe (1) | 70–90% granular population; 2 cases grade 3 and 1 case grade 4; all cases GFAP+, IDH−, ATRX retained | Molecular features compared to control series of high-grade astrocytomas c-Met tumour expression correlated with granular cell morphology  All GCAs showed Cav1/YKL-40/c-Met co-expression vs 4 cases in control cohort | 2 cases died of disease (7 months and 1 month); 1 case no evidence of disease at 10 months follow-up |
